# Prevalence of different types of vaginitis in Pap-smear samples from the Women’s Clinic of Firouzabadi Hospital in Tehran 2019 to 2021

**DOI:** 10.3205/dgkh000627

**Published:** 2026-02-17

**Authors:** Farahnaz Farzaneh, Fatemeh Montazer, Kaveh Khosravi

**Affiliations:** 1 Obstetrics and Gynecology Dept., School of Medicine, Iran University of Medical Sciences, Tehran, Iran; 2 Pathology Dept., School of Medicine, Iran University of Medical Sciences, Tehran Iran; 3 Medical Student, Faculty of Medicine, Iran University of Medical Sciences, Tehran, Iran

**Keywords:** Vaginitis, Pap smear, prevalence, etiology, vaginal atrophy, bacterial vaginitis, Trichomonas vaginalis, Candida albicans, inflammation, metaplasia

## Abstract

**Introduction:**

Vaginitis is one of the most common causes of referrals to gynecologists and obstetricians. It is characterized by symptoms such as itching, vaginal discharge, and redness. The three main causes of vaginitis are bacteria, various species of yeast and the protozoan *Trichomonas (T.) vaginalis*. *Candida albicans* has been considered as the main cause of vulvovaginitis in different regions of the world. The most common types of anaerobic bacteria in bacterial vaginitis are *Gardnerella vaginalis*, Bacteroidetes, Prevotella and Peptostreptococcus spp. In Iran to date, few population-based studies have been conducted in relation to vaginitis. As Pap-smear studies are conducted for investigating tissue changes in order to diagnose malignancy, these clinical tests are also appropriate sources for the evaluation of vaginitis. This is a cross-sectional, retrospective study to investigate different types of vaginitis based on its causative agent, and to examine its complications.

**Methods:**

All female patients between the ages of 21 and 75 who were referred to the Women’s Clinic of Firozabadi Hospital during the years 2019 to 2021 were included in this study. Demographic information, including age and sex, as well as information related to the type of infection, the degree of inflammation, the presence or absence of squamous metaplasia, and the presence or absence of vaginal atrophy, were extracted from the patient files in the archives of Firozabadi Hospital.

**Results:**

Out of the 2050 people studied, 165 (12.4%) had a positive Pap smear to diagnose vaginitis. The average age of the studied patients was 43. ±9.9 years; the age range was 21 to 72 years. Bacterial vaginitis (BV) was the most prevalent type (54.5%), the second-most frequent type was candidiasis vaginitis, and the least frequent type was caused by *T. vaginalis*.

**Conclusion:**

The high prevalence of BV indicates that vaginitis is a major problem in the clinic. The use of Pap smear is not only a useful indicator in cytology to detect premalignant changes, but it can also be a useful and practical method in diagnosing the type of vaginitis. Furthermore, Pap smears as a diagnostic method for vaginitis can help provide correct treatment, design prevention programs, strengthen the immune system, and prevent multiple vaginal flora changes.

## Zusammenfassung

**Einleitung:**

Vaginitis ist eine der häufigsten Ursachen für Überweisungen an Gynäkologen und Geburtshelfer, die durch Symptome wie Juckreiz, Scheidenausfluss und Rötung gekennzeichnet ist. Die drei Hauptursachen für Vaginitis sind Bakterien, Candida (C.) spp. und Trichomonaden. *C. albicans* gilt in verschiedenen Regionen der Welt als Hauptursache der Vulvovaginitis. Die häufigsten Arten anaerober Bakterien bei bakterieller Vaginitis sind *Gardnerella vaginalis*, Bacteroidetes, Provetella und Peptostreptococcus spp. Im Iran wurden bisher keine zahlreichen und umfassenden Studien zur Vaginitis durchgeführt, die sich auf die weibliche Bevölkerung unter verschiedenen Bedingungen und zu verschiedenen Zeiten beziehen. Da Pap-Abstriche zur Untersuchung von Gewebeveränderungen durchgeführt werden, um Malignität zu diagnostizieren, sind diese klinischen Tests geeignete Quellen auch für die Bewertung der Vaginitis.

**Methode:**

Es handelt sich um eine retrospektive Querschnittsstudie mit dem Ziel, verschiedene Arten der Vaginitis auf der Grundlage ihrer Erreger und ihrer Komplikationen zu untersuchen. Alle Patientinnen im Alter zwischen 21 und 75 Jahren, die in den Jahren 2019 bis 2021 in die Frauenklinik des Firozabadi Hospitals eingewiesen wurden, wurden in die Studie aufgenommen. Demografische Informationen einschließlich Alter und Geschlecht sowie Informationen über die Art der Infektion, den Grad der Entzündung, das Vorhandensein oder Fehlen einer Plattenepithelmetaplasie und das Vorhandensein oder Fehlen einer vaginalen Atrophie wurden den Patientenakten im Archiv des Firozabadi Hospitals entnommen.

**Ergebnisse:**

Von 2050 untersuchten Personen hatten 165 (12,2 %) einen positiven Pap-Abstrich zur Diagnose einer Vaginitis. Das Durchschnittsalter der untersuchten Patientinnen betrug 43,5±9,9 Jahre; die Altersspanne reichte von 21 bis 72 Jahren. Die höchste Häufigkeit betraf die bakterielle Vaginitis (BV) (54,5 %), die niedrigste die trichomonale Vaginitis.

**Schlussfolgerung:**

Die hohe Prävalenz der BV deutet darauf hin, dass die Vaginitis ein Problem in der Klinik darstellt, und die Candidiasis ist die zweithäufigste Art der Vaginitis, die im Pap-Abstrich nachgewiesen wurde.

Die Verwendung des Pap-Abstrichs ist nicht nur ein nützlicher Indikator in der Zytologie, um prämaligne Veränderungen zu erkennen, sondern ist auch eine nützliche und praktische Methode, um eine Vaginitis zu diagnostizieren und Behandlungs- und Präventionsprogramme anzubieten, das Immunsystem zu stärken und multiple Veränderungen der Vaginalflora zu verhindern.

## Introduction

Vaginitis is one of the most common causes of referrals to physicians, and the most common cause of referrals to gynecologists and obstetricians. It is characterized by symptoms such as itching, vaginal discharge, and redness. The three main causes of vaginitis are various species of bacteria, Candida spp., and the protozoan *Trichomonas (T.) vaginalis* [1], [2].

In the last decade, the prevalence of vaginitis has increased significantly, so that the proportion of non-albicans variants is increasing [3]. As *Candida (C.) albicans* has been considered as the main cause of vulvovaginitis in different regions of the world, other causes of vaginitis may have been ignored. About one-third of all reports of vulvovaginitis occur in sexually active women. About 70% of people who have suffered from candidiasis at some point were affected by C. albicans type. Recurrent vulvovaginitis is one of the most common problems of the infected patients; in other words, among the pathogens, C. albicans is currently the most common causative agent [4], [5], [6].

*T. vaginalis* is another cause of sexually transmitted diseases (STD) in men and women. This pathogen is located in the lower part of the urinary tracts of women and in the prostate and urinary tracts of men, which not only causes vaginitis and urinary diseases, but is also associated with other diseases such as HIV [7], [8].

Bacterial vaginitis (BV) is a vaginal condition in which the disease occurs with or without symptoms after disturbed equilibrium in the vaginal area between H_2_O_2_-producing lactobacilli and *Gardnerella (G.) vaginalis*. In general, the most common types of anaerobic bacteria in bacterial vaginitis are *G. vaginalis*, Bacteroidetes, Prevotella and Peptostreptococcus spp.

BV is a common problem in the reproductive system of women worldwide during their reproductive years and is related to infections due to different pathogens such as HIV and other sexually transmitted diseases. BV almost always recurs after treatment, and about 50% of women suffer from its complications for up to 12 months.

There are several factors associated with BV, including age, smoking, ethnicity, culture, socioeconomic status, having multiple sexual partners, and antibiotic therapy.

Early diagnosis of vaginitis and its appropriate and timely treatment is very important, because otherwise these infections can lead to serious complications, such as pelvic inflammatory diseases, infertility, chronic pelvic pain, premature birth and the risk of HIV infection. It is possible to diagnose the pathogenic factors of vaginitis through examining patients’ complaints, clinical examination and laboratory methods [9]; however, because of economical problems, it is not possible to easily perform these tests for most of the developing countries.

In addition, limited access to medical services, low level of awareness, and cultural barriers are among the reasons for delaying the treatment of this disease. Therefore, it is suggested that in order provide timely treatment and prevent serious complications of this disease, clinical examination and its combination with patients' complaints should be used.

Pap smear, which is a screening test to take samples from the cervix and examine them for abnormal cells that lead to cervical cancer. In addition, Pap smears can provide useful information regarding the presence of vaginitis in patients [10].

Cultural barriers and the difficulty of vaginal examinations in many cases lead to women to avoid gynecological visits, and sometimes treatment is only sought given complaints.

In Iran to date, numerous and extensive populational studies on vaginitis are largely lacking, or the studies have only been conducted cross-sectionally and in a limited population. On the other hand, Pap smear studies have been conducted with the aim of investigating tissue changes to diagnose malignancy. Vaginitis is usually not investigated in such studies, although such available samples are a very suitable source for checking for vaginitis, determining its prevalence, and identifying its various causal organisms (bacteria and yeast).

In addition, in such studies, the sensitivity and specificity of complaints and clinical tests along with laboratory diagnosis for the correct diagnosis of vaginitis have rarely been investigated [11].

For the optimal use of Pap smear in the analysis of vaginitis and to eliminate the limitations in the studies that were mentioned above, the frequency of vaginitis types in the Pap smear samples from the Women's Clinic of Firouzabadi Hospital in Tehran from 2019 to 2021 was investigated, as were its complications.

## Methods

### Study design

 This is a cross-sectional, retrospective study designed to determine the percentage of patients who had positive Pap smear for vaginitis, the percentage of patients with genital tract inflammation, the incidence of BV, the rate of candidal vaginitis (CV), the rate of trichomonal vaginitis, the relationship between age and the prevalence of vaginitis, and the relationship between the prevalence and the type of vaginitis.

After obtaining the necessary approval from the university’s ethics committee (code number IR.IUMS.REC.1401.361), the study was started.

### Study population

All female patients between the ages of 21 and 75 who attended to the Women’s Clinic of Firozabadi Hospital during the years 2019 to 2021 were included in this study. Inclusion criteria were:

- Being between the ages of 21 and 75 and visiting between 2019 and 2021,
- performing a Pap smear test at the Women’s Clinic of Firozabadi Hospital.

Exclusion criteria were:

- Incomplete data in the file,
- patient information not available,
- Pap smear samples were incomplete or insufficient.

### Sampling method

Sampling was done by census, so that all women attending to the women Department of Firozabadi hospitals between 2019 and 2021 were included.

Demographic information, including age, as well as information related to the type of infection, the degree of inflammation, the presence or absence of squamous metaplasia, and the presence or absence of atrophy, were extracted from the patient files in the archives of Firozabadi Hospital and were inserted in a researcher-made questionnaire, the validity and reliability of which was confirmed. The obtained information was analyzed using SPSS v.26 software. The results of quantitative variables were expressed as mean and standard deviation (mean±SD), and for qualitative variables as frequency and frequency percentage. The chi-squared test was used to compare two qualitative variables. A significance level of p<0.05 was set.

## Results

Out of the 2,050 women studied, 165 women (12.4%) had a positive Pap smear to diagnose vaginitis. The average age of the studied patients was 43.5±9.9 years and the age range was 21 to 72 years. The highest frequency was related to BV (54.5%), and the lowest frequency was related to trichomonal vaginitis (Table 1 (Tab. 1)).

During the 3-year study period, an upward trend was apparent in the frequency of both BV and CV. No cases of trichomonal vaginitis were reported during these three years. Only one case of simultaneous bacterial and candidal vaginitis was identified in 2019 (Table 2 (Tab. 2)).

The mean age for BV was significantly higher than that of CV (Table 3 (Tab. 3)).

Only in 15 out of 165 samples (9.1%) was vaginal atrophy. In the Pap smear samples examined in patients with CV, 2.7% exhibited vaginal atrophy, while in Pap smear samples with BV, 14.4% atrophy of vagina was reported. There was only one sample of simultaneous bacterial and candidal vaginitis, and no vaginal atrophy was reported in the microscopic examination of this sample (Table 4 (Tab. 4)).

The average age of the patients whose Pap smear sample was positive for vaginal atrophy was 56.6 years, and for the negative samples, it was 42.9 years.

About 57.5% of the examined samples had mild inflammation, 35.7% showed moderate and only 6.8% had severe inflammation. 56.7% of patients with CV had moderate inflammation and only 9% had severe inflammation, while 78.8% of cases with BV had mild inflammation and only 2.4% showed severe inflammation. The only patient with simultaneous candidal and bacterial vaginitis had mild inflammation (Table 5 (Tab. 5)).

63% of the examined samples had no positive results for squamous metaplasia. 34.4% of patients with CV were positive for squamous metaplasia, while 41.1% of patients with BV were positive for squamous metaplasia (Table 6 (Tab. 6)).

## Discussion

In this study, patients were evaluated who attended the Women's Clinic of Firouzabadi Hospital between 2019–2021 for whom a Pap smear was performed. Cervical cytology is one of the most widely used methods and is mostly known for the cytological changes seen in precancerous lesions and invasive cervical cancer [12]. Various infectious conditions lead to changes in the appearance of benign squamous and glandular cells in cervical cytology samples. A variety of physiological and pathological conditions are responsible for the transformation of the multi-microbial flora of the vagina into mono-microbial, in which pathogenic microbes may grow excessively and lead to inflammation of the cervix and vagina.

Chronic irritation of the cervix due to intrauterine devices, chemical stimuli, inflammation or infection, endocrine changes and repair changes can lead to alarming parakeratosis, hyperkeratosis and squamous metaplasia of the non-keratinized squamous mucosa of the cervix and vagina [12]. Vaginal and cervical mucosa are very similar; therefore, both are at the same risk of infection. Many infectious organisms that cause inflammation are sexually transmitted. Good general health status, an adequate immune system, intact squamous epithelium, acidic vaginal PH, and a balance between the various microorganisms that usually coexist are responsible for natural protection against various infections [13]. Therefore, vaginal infections of external origin or pathogenicity of the vaginal microbiota occur when the squamous epithelium of the vagina and cervix is not fully mature (preadolescent girls), its thickness is reduced (menopausal women), or when the epithelium is damaged during pregnancy or childbirth [14]. 

The current study showed that BV had the highest prevalence rate compared to CV and *Trichomonas vaginitis*. The high prevalence of BV indicates that vaginitis is a major problem in the clinic and requires more effective diagnostic and treatment methods. Therefore, prompt identification and treatment of BV is critical to prevent complications and improve reproductive health. Candidiasis, which is related to an abnormal overgrowth of Candida spp., is the second most common type of vaginitis detected in Pap smears. Candida infections are usually accompanied by itching, discharge and discomfort in the vagina. Although candidiasis is generally recognized as a common and self-limiting infection, it can significantly affect a person’s quality of life. Considering the high prevalence of candidiasis, the need for accurate diagnosis and appropriate anti-candida treatment to reduce symptoms and prevent frequent infections is obvious. Trichomoniasis is the least common of the three types of vaginitis. However, this should not diminish its importance, as trichomoniasis is a sexually transmitted infection with adverse consequences for reproductive health. In our study, no cases of trichomonas vaginitis were identified, but this could be due to the errors and limitations of the laboratory, sampling, and design implementation method.

Early diagnosis of trichomoniasis in Pap smear samples should lead to immediate and appropriate treatment to prevent transmission of infection to sexual partners and prevent possible adverse consequences, such as infertility and increased vulnerability to other sexual infections. Our findings emphasize the importance of screening and diagnosis of vaginitis in the clinical environment. Pap smears are widely used to screen for cervical cancer, but they can also provide valuable information about the presence of vaginal infections. Integrating additional diagnostic tests, such as liquid microscopic methods, or nucleic acid amplification tests, into routine screening protocols can increase diagnostic accuracy and improve patient outcomes. 

The prevalence of vaginitis was reported by Ayatollahi et al. [13] as 25.3%, by Danesh et al. [15] as 32%, by Cheraghi et al. [16] as 8%, by Hezarjaribi et al. [17] as 10.6%, and in Nepal as 29.1% [14]. The prevalence of 12.4% in our study falls within midrange of those other studies. All the studies above indicate the necessity of investigation in different communities. 

The change in the prevalence of vaginitis and its different types is related to geographical changes and sexual behaviors, culture and customs of different regions of the country, as well as demographic differences and diagnostic methods in clients. In the study by Ayatollahi et al. [13], the average age was 31, in the study of Yazd [15], the average age was 30 to 39 years, and in the study of Kalantari et al. [18], the average age was 20 to 30 years, while in our study the average age was 43.5 years. Therefore, it again emphasizes on the importance of investigating the infection in different regions and different ages. 

In the study by Danesh et al. [15], the prevalence of BV was 29%, CV 4.25%, and trichomonas vaginitis 0.37%, while in another study, the prevalence of BV was 12.1% and CV was 5.8% [19]. In the study by Ziaei et al. [20], the prevalence of BV was reported as 46.1%, CV 46.6%, and trichomoniasis 7.3%, which agree with the results of our study in finding the highest prevalence for BV. However, the results of our study show the similarity in the prevalence of CV and BV moreso than other studies.

In various studies, the relationship between the level of education and bacterial infection has been proven. However, no correlation has yet been reported between the marital status, husband’s job and wife's job with the prevalence of vaginitis. The study by Ziyai et al. [20] have shown a significant relationship between age and the type of vaginitis. In our study, the type of vaginitis was reported to be different at different ages: that BV is more common in older women. 

An interesting finding of our study is the increasing prevalence of vaginitis with time: the prevalence of CV and BV in 2021 was twice as high as the prevalence of these infections in 2018. This might be due to improvement in the diagnostic methods including the Pap smear test, so that it was possible to detect these infections more accurately. However, it is important to bear in mind that sexual behavior, population density and other factors also affect the prevalence. It is estimated that 50 million cases of vaginal squamous intraepithelial lesions are discovered every year worldwide [21]. Aerobic vaginitis and BV can be signs of risky sexual behavior and exposure to HPV. An important research question is whether changes in normal vaginal flora can indicate the presence of high-risk HPV infection and whether aerobic and BV are associated with squamous metaplasia. The results of the present research have useful information in this field, as 37% of the Pap smear samples examined in the patients were positive for squamous metaplasia, and this can be a confirmation of the relationship between infections and metaplasia.

The findings that 34.4% of patients with Candida-based vaginitis and 41.1% of patients with BV had squamous metaplasia, and 2.7% of patients with CV and 14.4% with BV showed vaginal atrophy in their Pap smear samples, highlight the importance of focussing more attention on this issue. In other words, BV has caused more metaplasia and vaginal atrophy in patients, and this itself proves the connection between infection and residual complications [19], [20], [21], [22], [18]. Increasing awareness about the importance of seeking early treatment and safe sexual behaviors can help reduce the burden of these infections. In addition, the implementation of appropriate treatment guidelines and providing access to effective drugs at reasonable prices are essential steps in the management and control of this type of vaginitis.

## Conclusions

Analysis of Pap smear samples showed a high prevalence of BV and CV. The use of Pap smear is not only a useful indicator in cytology to detect premalignant changes, but it can also be an effective and practical method for diagnosing the type of vaginitis and providing treatment and prevention programs, improving the personal health and preventing multiple vaginal flora changes. These findings show the need for routine screening, accurate diagnosis and rapid treatment of vaginitis in clinical practice. The high prevalence of BV requires effective management strategies to prevent complications and improve health status.

CV, although common, should not be ignored because it can have a significant impact on the quality of life of involved people. Trichomoniasis, despite its low prevalence, requires immediate attention and appropriate treatment to prevent transmission and possible complications. Public health interventions should focus on increasing awareness, services, and continuing education of both health care providers as well as patients, in addition to ensuring access to the correct diagnostic and therapeutic tools and options. By addressing these issues, we can effectively reduce the burden of vaginitis and improve the overall health and well-being of women in the clinic's patient population.

## Notes

### Competing interests

The authors declare that they have no competing interests.

### Ethical approval 

This study was conducted after approval by the ethics committee of the University of Medical Sciences, Tehran, Iran (internal registration number IR.IUMS.REC.1401.361).

### Funding 

None.

### Acknowledgment 

Many thanks for all the support and assistance of all those working in different Departments of the Women’s Clinic at Firouzabadi Hospital. 

### Authors’ ORCIDs

- Farzaneh F: https://orcid.org/0000-0002-2422-133X
- Montazer F: https://orcid.org/0000-0002-4487-1399
- Khosravi K: https://orcid.org/0009-0005-3329-5743

## Figures and Tables

**Table 1:**
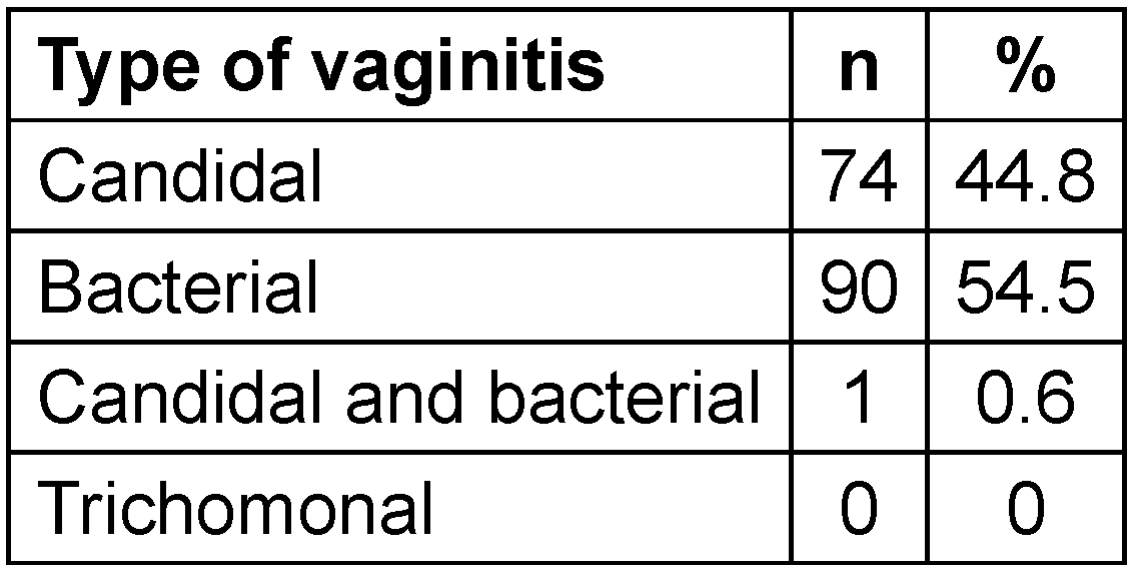
Types of vaginitis based on its causative factor

**Table 2:**
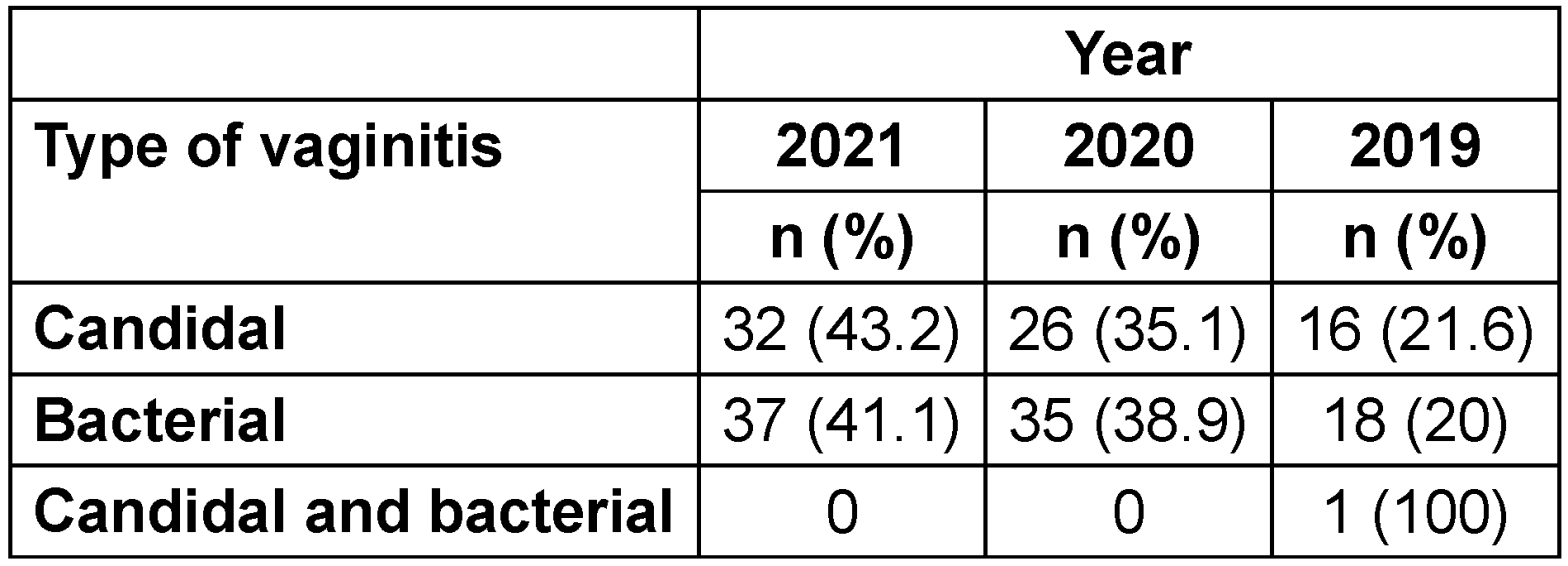
Prevalence of vaginitis based on the year of study

**Table 3:**
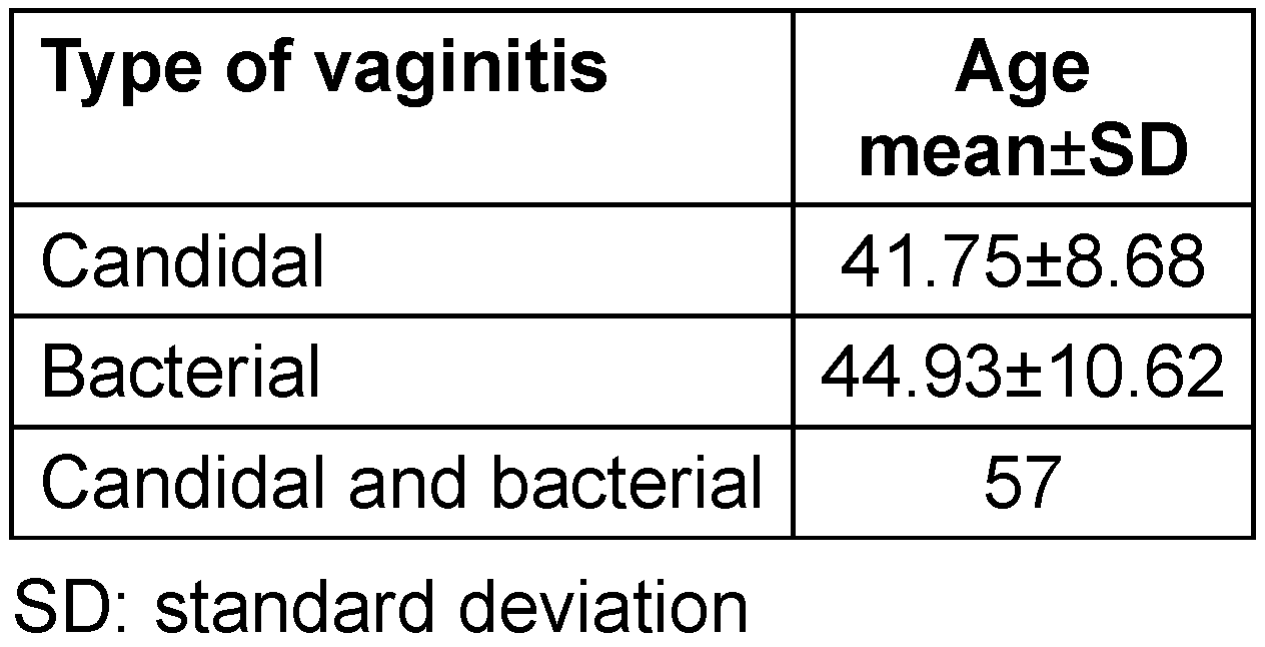
Mean age in different types of vaginitis

**Table 4:**
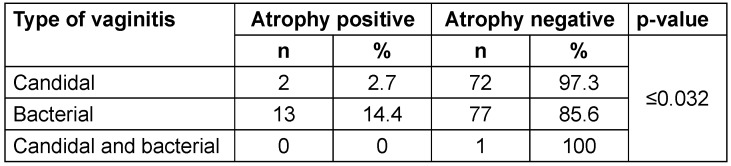
Frequency of vaginal atrophy among patients with vaginitis of different etiologies

**Table 5:**
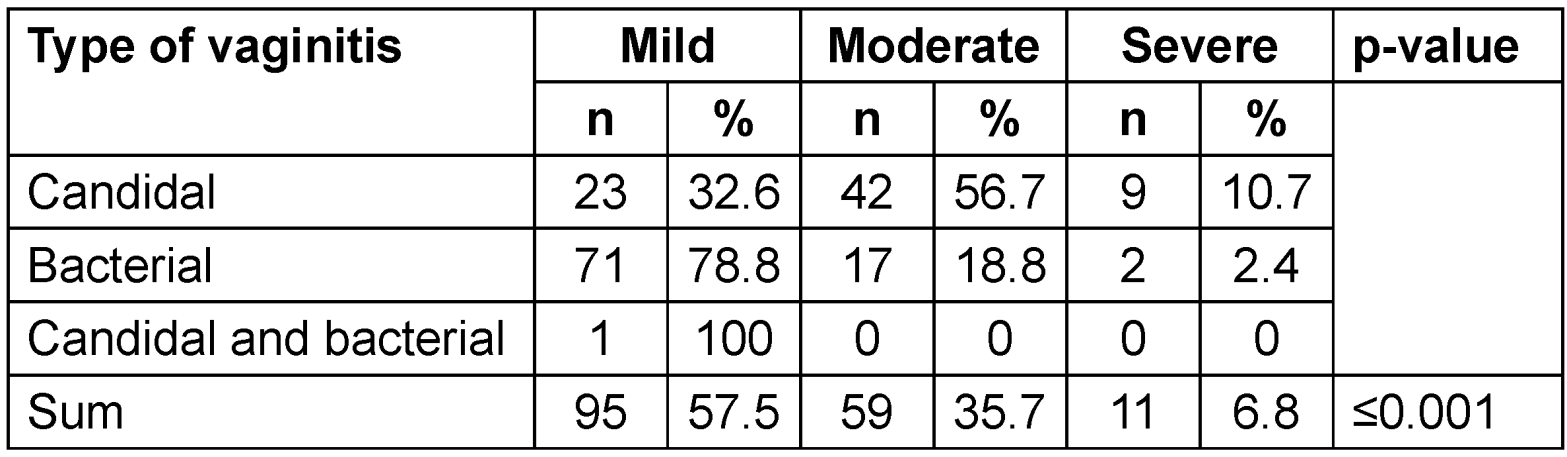
Prevalence of inflammation severity in the examined samples according to the type of vaginitis

**Table 6:**
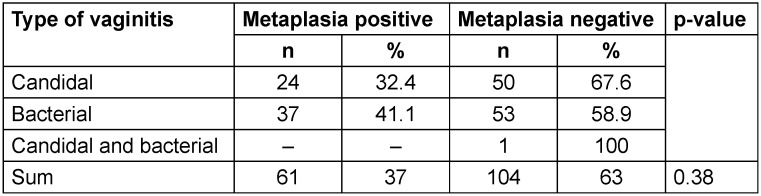
Frequency of squamous metaplasia among different types of vaginitis
